# Stepping up: impact of health education on diabetic foot Self-Care in a Resource-Limited tertiary care setting

**DOI:** 10.1038/s41598-025-08246-1

**Published:** 2025-07-08

**Authors:** Aya Mohamed Mehana, Amal Selim, Shimaa M. Saied, Nadira Mansour Hassan

**Affiliations:** 1 Department of Public health and Community Medicine, Faculty of medicine, Tanta University, Tanta City, Egypt; 2 Department of Internal Medicine, Faculty of Medicine, Tanta University, Tanta City, Egypt

**Keywords:** Awareness, Practices, Foot self-care, Diabetic, Health education, Intervention, Diseases, Endocrinology, Health care

## Abstract

Up to 15% of patients with diabetes worldwide experience foot problems, which are the most frequent cause of their hospitalization. Proper foot care is essential to avoid these problems and enhance the patient’s quality of life. This study aimed to assess the effectiveness of a health education intervention on awareness and practice of foot self-care among diabetic patients. This study was conducted at the “Endocrinology Outpatient Clinic” of Tanta University Hospitals, Egypt. A hundred diabetic patients were recruited for the study and then divided into intervention and control groups. A baseline assessment of foot self-care awareness and practice was done for both groups using a structured questionnaire. The control group received their routine care provided by the clinic, while the intervention group received an additional health education program. All patients were reassessed after three months. In the intervention group, the total awareness score was improved from (9.49 ± 1.65) preprogram to (13.70 ± 2.37) post-program (*p* < 0.001). Also, the total score of practice was improved from (38.15 ± 6.30) preprogram to (53.47 ± 6.03) post-program (*p* < 0.001). The total score of awareness and practices revealed a significant positive correlation (*r* = 0.59). A large effect size was found for both after the intervention. The study demonstrated a notable lack of awareness and adherence to foot self-care measures among patients with diabetes, which were markedly improved after implementing a foot self-care educational program. So, a health education program must be incorporated as special sessions at outpatient clinics for patients with diabetes to lower the risk of diabetic foot problems in Egypt.

**Supplementary Information:**

The online version contains supplementary material available at 10.1038/s41598-025-08246-1.

## Introduction

Diabetes mellitus (DM) is a serious public health issue that is becoming more prevalent worldwide. The global prevalence of diabetes was estimated to be 9.3% (463 million people) in 2019, rising to 10.2% (578.4 million) by 2030 and 10.9% (700.2 million) by 2045, according to the International Diabetes Federation Atlas in 2022^1^. Diabetes is the primary cause of acute coronary syndrome, lower limb amputation, blindness, chronic kidney failure, and stroke in Egypt^2^.

Diabetics with foot ulcers (DFUs), peripheral vascular disease, and or lower limb diabetic neuropathy are at risk for developing diabetic foot disease (DFD). Many factors could trigger this condition, but the most frequent ones include trauma, foot deformities, pressure on the foot, peripheral vascular disease, and peripheral neuropathy^3^. At the time of diagnosis of type 2 diabetes mellitus, over 10% of patients already have one or more of these risk factors^4^.

Globally, the prevalence of DFD is 6.3%, with a higher prevalence in type 2 diabetes compared to that in type 1^5^. In Egypt, a study conducted in 2021 revealed that 6.1–29.3% of diabetes patients have DFUs^6^. Nearly half of all diabetic patients will be affected by DFD throughout their lives, and it is responsible for 80% of all non-traumatic lower-limb amputations^7^. The most efficient way to prevent diabetic foot disease and amputations is by increasing awareness, adopting a good daily foot care practice, and ensuring good glycemic control in diabetic patients^8^. The American Diabetes Association advised that all diabetics should be educated on self-foot care to increase their knowledge and encourage good practices^9^.

Identifying the awareness gap and providing health education programs could improve diabetic patient practices, quality of life, and health outcomes. So, this study aimed to estimate the effectiveness of health education intervention on the awareness and practice of foot self-care among diabetic patients receiving care at Tanta University Hospitals.

## Methods

### Study design, setting, and duration

This randomized controlled study was conducted at the “Endocrinology Outpatient Clinic” of the Internal Medicine Department, Tanta University Hospitals, from November 2021 to October 2022. Tanta City is the capital of Gharbia Governorate in the Middle Delta region of Egypt. It is 92 Km north of Cairo. They are tertiary hospitals with an inpatient bed capacity of 1962 beds and 23 outpatient clinics. The Endocrinology Outpatient Clinic is the largest referral clinic for endocrine diseases in the Gharbia governorate. It provides free service and receives cases from approximately 5 million inhabitants. This unit is open 4 days a week with a rate of (200–250) patients per month. Regular follow-up for patients is routine in this clinic.

### Study population

This study targeted diabetic patients attending the “Endocrinology Outpatient Clinic” of the Internal Medicine Department, Tanta University hospitals, for treatment and follow-up. The sample size was calculated using the Epi Info software statistical package prepared by the WHO and the CDC. The required sample size was 86 patients, subsequently increasing by 20% to 100 to accommodate anticipated attrition. A random sampling method was employed to select 100 eligible patients from the target population. These individuals were then randomly allocated to either an intervention group or a control group using a concealed randomization procedure. The randomization sequence was generated using Microsoft Excel’s random number generator. participants were assigned using simple randomization without blocking or stratification.

### Inclusion criteria

Adult patients (> 18 years) previously diagnosed with diabetes mellitus type I or II attending the “Diabetic Outpatient Clinic” of the Internal Medicine Department in Tanta University Hospitals and agreed to be interviewed again after 3 months, were included in this study.

### Study tool and data collection

Data was collected by interviewing patients using a structured questionnaire specially designed after a thorough literature review^10,11^, it was used before and three months after program implementation, and it was divided into four main sections. The 1 st section included the sociodemographic data of the patients. The 2nd section contained the illness-related data of the patients as type of diabetes, duration, type of treatment, diabetic complications, and comorbidities. The 3rd section contained questions regarding the awareness of the patients about daily foot care. The 4th one aimed to assess the practice of daily foot care via 21 questions about foot inspection, shoe inspection before putting on, washing feet with warm water, drying feet after washing, using emollients for dry skin, cutting toenails properly, walking barefoot, wearing shoes without socks, check between toes, drying between toes, checking water temperature before bath and seeking professional help for any problem. The content validity was done through 4 panels of experts, all of whom were professors of public health and community medicine, and their opinions were requested via an assessment form. Modifications were carried out according to the experts’ judgment on the clarity and content appropriateness. The structured awareness section had a percentage of 93% while the practice section had 90%. Alpha Cronbach’s reliability was 0.976.

### Scoring system

In the awareness section, each correct answer scored one degree, and each incorrect answer scored zero. The total score was 18 degrees. awareness was considered poor if below 50%, average from 50 to 70%, and good if more than 70% of the total score. while in the practice section, the responses were rated on a five points Likert scale and answered by (always, often, sometimes, rarely, and never) each of them took (4, 3, 2, 1, and 0) respectively except in questions (wearing sandals, slippers or pointed toed shoes/wearing shoes without socks/walking barefoot) the score was reversed. The total score was 84 degrees. Practice was considered unsatisfactory if (< 60%) and satisfactory if ≥ 60% of the total score.

### The study procedure

Patients of both groups were exposed to a baseline assessment of foot self-care awareness and practice. The intervention group received an additional health education program on foot care behavior, while the control group received routine care only. All patients were reinterviewed after three months to assess the improvement in foot self-care awareness and practices.

**Content of the health education program**: The program contained information based on the Centers for Disease Control (CDC) Guidelines^12^. The first session introduced the various types of diabetes, their chronic complications, risk factors, and the potential complications associated with diabetic feet. The second session emphasized the significance of maintaining a controlled diet, adhering to a regular regimen of anti-diabetic medication, quitting smoking, and achieving glycemic control as essential measures in preventing the development of diabetic foot. Additionally, the session covered self-care practices for diabetic feet, including proper examination techniques for both feet and footwear. Lastly, the session highlighted the alarming signs that warrant immediate attention.

### Conduction of the program

The program was conducted via educational sessions and small group discussions using lectures, posters, videos, and instructional booklets in Arabic. The intervention group patients were divided into five groups, each group including 8–10 patients. Then they were exposed to two sessions, each of which was repeated for each group separately. The first session was carried out during the baseline assessment phase, while the second session was held one week after the first one. Each session took about 25 to 30 min. The educational sessions were delivered by public health professionals and internal medicine physicians trained in diabetic foot care education using standardized materials based on the CDC guidelines.

### Ethical considerations

Before carrying out the study, official permission was obtained from the head of the internal medicine department and the head of Tanta University Hospital. The study was consistent with the ethical standards of the Ethical Committee of Scientific Research of Faculty of Medicine, Tanta University, Federal Wide Assurance (FWA00022834), (IRB 0010038), and approved (reference number 34735/6/21). Also, informed consent was obtained from patients, and the study reassured them about the confidentiality of any information obtained, and the results will only be used for research.

### Statistical analysis of data

Five patients were excluded from the study as they were lost during the follow-up period. Two were from the control group, and the other three were from the intervention group. These participants were excluded from the post-intervention analysis. The study didn’t apply intention-to-treat analysis; only those who completed their follow-up assessment were included in the outcome evaluation. Firstly, data entry was done in an Excel spreadsheet sheet then the data was exported to the Statistical Package for Social Sciences (SPSS) program version 26 for Sorting, tabulation, and analysis of data. Chi-square (χ2),

McNemar’s chi-square test, and Wilcoxon Signed Ranks Tests were used. P value ≤ 0.05 was considered significant.

Cohen d effect size of intervention was calculated for both awareness and practice of foot self-care from the following equation: d = (M1 - M2)/SD pooled.

where:

- M1 is the mean of the first group.
- M2 is the mean of the second group.
- SD pooled is the pooled standard deviation.

The results were interpreted as follows (d = 0.0–0.19 trivial effect, d = 0.20 small effect, d = 0.50 medium effect, d = 0.80 or higher large effect)^13^.

## Results

**Table 1:** Distribution of both studied groups regarding their sociodemographic characteristics.

Sociodemographic characteristics	Control group(*n* = 48)		Intervention group(*n* = 47)		χ^2^	*P*
	*n*	%	*n*	%		
Age in years						
< 40	16	33.3	14	29.8	2.233	0.327
40 -	23	47.9	18	38.3		
≥ 60	9	18.8	15	31.9		
**Sex**						
Male	24	50.0	28	59.6	0.879	0.412
Female	24	50.0	19	40.4		
**Residence**						
Urban Rural	18	37.5	22	46.8	0.844	0.409
	30	62.5	25	53.2		
**Educational level**					6.567	0.087
Illiterate	23	47.9	19	40.4		
Basic education	15	31.3	11	23.4		
Secondary/technical	6	12.5	11	23.4		
University	4	8.3	6	12.8		
**Occupation**						
Not working	16	33.3	14	29.8	0.138	0.71
Working	32	66.7	33	70.2		
**Income**						
Insufficient	19	39.6	26	55.3	MC 5.674	0.058
Sufficient	22	45.8	20	42.6		
Sufficient and saving	7	14.6	1	2.1		
**Family history of Diabetes**						
Negative	17	35.4	22	46.8	1.273	0.259
positive	31	46.6	25	53.2		

*SD: Standard Deviation χ*^*2*^: *Chi Square Test*.

t: Independent Sample T Test MC: Monte Carlo Exact Test.

Table 1 shows that there was no statistically significant difference between the control and the intervention group regarding sociodemographic characteristics.

**Table 2:** Distribution of the studied groups according to their illness-related data.

Illness-related data	Control group(*n* = 48)	%	Intervention group(*n* = 47)	%	χ2	P value
	n		n			
**Type of Diabetes**					0.407	0.524
Type I	14	29.2	11	29.2		
Type II	34	70.8	36	70.8		
**Duration of Diabetes**					1.927	
< 5 years	6	12.5	11	23.4		0.382
5-<10 years	19	39.6	16	34		
≥ 10 years	23	47.9	20	42.6		
**Treatment**						0.560
Insulin	14	29.2	14	29.8	MC 1.273	
Oral hypoglycemics	17	35.4	21	44.7		
Both	17	35.4	12	25.5		
**Comorbidities**	**(n = 41)**		**(n = 38)**		MC 2.794	0.610
Hypertension	30	62.5	28	59.8		
Hepatic	4	8.3	7	14.8		
Renal	3	6.3	1	2.1		
Cardiac	4	8.3	2	4.2		
**Complications of Diabetes**	**(n = 42)**		**(n = 37)**		MC 4.067	0.261
Neuropathy	27	56.3	25	53.2		
Nephropathy	8	16.6	10	21.3		
Retinopathy	7	14.6	2	4.2		
**Foot ulcer**					1.378	0.590
No	26	54.2	31	66.0		
Was present and healed	18	37.5	13	27.7		
Currently present	4	8.3	3	6.4		
**Regularly monitoring blood glucose**					0.562	0.755
No	15	31.3	12	25.5		
Yes	33	68.7	35	74.5		
**Previously advised about foot self-care**					3.622	0.310
**No**	32	66.7	27	57.4		
**Yes**	16	33.3	20	42.6		
In the last 3 months	4	25.0	9	45.0		
In the last 6 months	6	37.5	8	40.0		
In the last year	6	37.5	3	15.0		
**Source of the advice**	**(n = 16)**		**(n = 20)**		4.593	0.346
Media	3	18.8	6	20.1		
Physician	5	31.3	16	53.3		
Nurse	3	18.8	1	3.3		
Friend	3	18.8	3	10.0		
Family member	2	12.5	4	13.3		

**χ**^**2**^: Chi-Square Test MC: Monte Carlo Exact test.

Table 2 reveals that there was no statistically significant difference between the control and intervention groups regarding illness-related data.

**Table 3:** Comparison between both control and intervention groups regarding scores of foot self-care awareness and practice before and 3 months after program implementation.

Score	Foot self-care				t-test (P value)	
	Control group(*n* = 48)		Intervention group(*n* = 47)		Preprogram	Post program
	Baseline assessment	After 3 months	Preprogram	post -program		
**Awareness score**					1.305 0.195^a^	10.364 (< 0.001*) ^b^
Mean ± SD	9.02 ± 1.84	8.88 ± 2.17	9.49 ± 1.65	13.70 ± 2.37		
Range	5–13	4–14	6–14	8–17		
Median	9	9	9	14		
**Paired t-test**	0.414 (0.681)		13.394 (< 0.001*)			
**(P value)**						
**practice score**					1.805 0.074^c^	10.709 (< 0.001*) ^d^
Mean ± SD	36.04 ± 6.77	36.39 ± 6.73	38.15 ± 6.30	53.47 ± 6.03		
Range	28–53	28–58	29–60	45–68		
Median	34.5	34.5	39	51		
**Paired t-test**	1.470 0.148		13.921 (< 0.001*)			
**(P value)**						

SD: Standard Deviation *: statistically significant.

a: preprogram awareness in the control group vs. preprogram awareness in the intervention group.

b: post-program awareness in the control group vs. post-program awareness in the intervention group.

c: preprogram practice in the control group vs. preprogram practice in the intervention group.

d: post-program practice in control group vs. post-program awareness in the intervention group.

Table (3) shows that the **mean score of foot self-care awareness** initially among the control group shows a slight non-significant decrease from (9.02 ± 1.84) to (8.88 ± 2.17) after three months (*p* > 0.05). While in the intervention group, the mean awareness score was significantly improved from (9.49 ± 1.65) preprogram to (13.70 ± 2.37) three months post-program, with a p value < 0.001.

**Regarding foot self-care practice**, its mean score nearly did not change in the control group (36.04 ± 6.77 vs. 36.39 ± 6.73) initially and after three months, respectively. However, in the intervention group, the mean score of practice was markedly improved from (38.15 ± 6.30) preprogram to (53.47 ± 6.03) post-program with a statistically significant difference between both means (*p* < 0.001).

**Table 4:** Correlation between foot self-care awareness and practice after program implementation in intervention group patients.

(*N* = 47)	The total score of awareness	
Total score of practice	r	0.594
	p	< 0.001*

r: Pearson correlation coefficient *: statistically significant.

Table 4 illustrates that there was a significant (*p* < 0.001) positive moderate correlation (*r* = 0.59) between the total score of foot self-care awareness and that of practice in intervention group patients after program implementation.

**Table 5:** Effect size of intervention on foot self-care awareness and practice in studied groups.

Foot self-care	Cohen’s d effect size (d)
**Awareness**	2.13
**Practice**	2.51

(d = 0.0–0.19 trivial effect, d = 0.20 small effect, d = 0.50 medium effect, d = 0.80 or higher large effect).

Table 5 pointed out that the effect size of intervention on foot self-care awareness and practice was large (2.13, 2.51), respectively, and this indicates the effectiveness of the health education program.

## Discussion

Diabetes mellitus (DM) is a significant public health issue, with its chronic complications being a major contributor to increasing mortality and morbidity rates. One of the most prevalent complications is diabetic foot ulcers^14^. The most effective prevention strategy involves two key components: educating patients about diabetic foot complications and prevention and promoting healthcare by ensuring regular follow-up and check-ups for diabetic patients^15^. This study aimed to evaluate the impact of a health education intervention on diabetic patients’ awareness and practices related to foot self-care.

The present study results showed that there were no significant statistical differences between the control & intervention groups, revealing the homogeneity among study subjects regarding their characteristics, with about two-thirds of patients residing in rural areas, and illiteracy was predominant among nearly half of the studied patients of both groups. These findings were consistent with El***-Sedawy et al.***,*** (2016)***^16^. While it was inconsistent with ***Ali M et al.***,*** (2019)***^17^ who found that most participants lived in urban areas and around 30% had higher education.

Regarding illness-related data, there were also no statistically significant differences between the intervention and control groups. Around half of the participants in both groups had a family history of diabetes, and over 80% of patients in both groups had comorbid conditions, with hypertension affecting nearly two-thirds. Most patients in both groups were on oral hypoglycemic medications, and 56.3% of the intervention group and 53.2% of the control group experienced foot numbness. These findings are consistent with those of ***Sharoni S et al.***,*** (2016)***^18^ who stated in their study that most of the patients were on oral medications (74.2%) and had comorbid diseases (93.5%). ***Moussa M et al.***,*** (2017)***^19^ similarly found that 70% of their participants had a family history of diabetes and 91.7% had comorbidities such as hypertension (63.6%).

Concerning awareness and practice levels, the study found that the mean score for foot self-care awareness and practices improved significantly among patients in the intervention group after the educational program, while there was no significant change in the control group. The effect size of the intervention on awareness and practice was notably larger in the intervention group.

The large effect sizes observed in awareness and practice may be attributed to several factors. Firstly, patients had low baseline knowledge, and the practices allowed for greater relative improvement. Secondly, the intervention was delivered through small interactive groups using culturally appropriate materials which likely enhanced engagement and retention. Finally, the structured design and short-term follow-up likely captured the peak of behavioral change before any decline could occur.

The findings of this study are consistent with international literature ***Grillo M et al.***,*** (2016)***^20^ in Brazil demonstrated that structured diabetes education significantly improved patients’ knowledge and behaviors. likewise, ***Singh et al. (2020)***^21^ in India showed enhanced food care practices were shown following culturally adapted educational programs. These parallels suggest that interactive, tailored education, when delivered in small groups using accessible materials, can produce meaningful behavioral changes across diverse populations. This underscores the adaptability and the effectiveness of such programs in resource limited settings like Egypt.

Moreover, a systematic review and meta-analysis by ***Yıldırım Ayaz et al.***,*** (2022)***(22) showed that diabetic foot education positively impacted patients’ knowledge and behaviors. They highlighted that after amputation, patients’ 5-year mortality rate ranged from 40 to 90%, with most of these complications being preventable through annual foot examinations and routine foot care, achievable via regular educational programs. These findings, supported by earlier studies, reinforce the effectiveness of foot self-care education programs, showing that when patients simply receive information, their knowledge improves, which positively affects their practices.

Additionally, the intervention group in the current study showed a significant positive correlation (*r* = 0.59) between foot self-care awareness and practice after implementation of the program. This is consistent with ***Alharbi and Sulaiman***,*** (2022) study***(23) which also emphasized that patients with better knowledge had better foot-care practices. Along the same line, the most important aspect of ***Khan et al. (2020)***(24) study is the positive correlation between the foot-care knowledge score and practice score.

Diabetic foot education conducted in a structured and organized manner as part of the multidisciplinary management of patients with diabetes plays an important preventive role.

## Conclusion and recommendations

The study demonstrated a notable lack of knowledge and adherence to foot self-care measures among diabetic patients. To address this deficiency, implementing standardized foot self-care educational programs within diabetic outpatient clinics is strongly recommended. These programs should be facilitated by a certified diabetic educator in conjunction with a podiatrist to optimize foot screening procedures and foster patient education regarding foot health.

## Strengths and limitations of the study

This study effectively investigated the impact of a comprehensive educational program on improving foot self-care practices and awareness among diabetic patients. However, the study’s single-center design limits the generalizability of its findings. Additionally, the reliance on self-reported data and loss of follow-up could introduce biases. Moreover, the short follow-up period limited the ability to assess the long-term sustainability of improvements in awareness and practice. Future studies with longer durations are recommended to evaluate lasting behavioral changes. This study was not registered in a clinical trial registry, which limits the transparency and replicability of findings.

## Electronic supplementary material

Below is the link to the electronic supplementary material.

Supplementary Material 1

## Data Availability

Data is provided within the manuscript or supplementary information files.

## References

[1] Ogurtsova, K. et al. Global estimates of undiagnosed diabetes in adults for 2021. *Diabetes Res. Clin. Pract.* **183** (8), 109–118 (2022). 10.1016/j.diabres.2021.109118 34883189

[2] Hegazi, R., El-Gamal, M., Abdel-Hady, N. & Hamdy, O. Epidemiology of and risk factors for type 2 diabetes in Egypt. *Annals Global Health*. **81** (6), 814–820 (2015). 10.1016/j.aogh.2015.12.011 27108148

[3] Tuttolomondo, A., Maida, C. & Pinto, A. Diabetic foot syndrome: Immune-inflammatory features as possible cardiovascular markers in diabetes. *World J. Orthop.* **6** (1), 62–76 (2015). 25621212 10.5312/wjo.v6.i1.62 PMC4303791

[4] Abouzid, M. R., Ali, K., Elkhawas, I. & Elshafei, S. M. An overview of diabetes mellitus in Egypt and the significance of integrating preventive cardiology in diabetes management. *Cureus* **14** (7), 27–66 (2022). 10.7759/cureus.27066 PMC9390800 36000101

[5] Zhang, P. et al. Global epidemiology of diabetic foot ulceration: A systematic review and meta-analysis. *Ann. Med.* **49** (2), 106–116 (2017). 27585063 10.1080/07853890.2016.1231932

[6] Galal, Y. S., Khairy, W. A., Taha, A. A. & Amin, T. T. Predictors of foot ulcers among diabetic patients at a tertiary care center, Egypt. *Risk Manage. Healthc. Policy*. **14**, 3817–3827 (2021). 10.2147/RMHP.S325065 PMC8458748 34566439

[7] Kassab, H. S., Ismaeal, M. T., Abd Elfattah, T. & Elaaty, A. Diabetic foot care knowledge and practice in type 2 diabetes and relation to microvascular complications in Alexandria (Egypt). *Endocr. Regul.* **56** (2), 95–103 (2022). 35489046 10.2478/enr-2022-0011

[8] Saidi, S. B., Jabar, J. A. & Firdaus, M. K. Determining knowledge and practice of self-foot care and adherence of patients on self-foot assessment among patients with diabetes mellitus. *Malaysian J. Nurs.* **14** (4), 62–72 (2023).

[9] Sari, Y. et al. Foot self-care behavior and its predictors in diabetic patients in Indonesia. *BMC Res. Notes*. **13** (1), 1–6 (2020). 32005281 10.1186/s13104-020-4903-y PMC6995081

[10] Mehmood, M. K. et al. Diabetic foot self-care: awareness and practice among type 2 diabetic patients in primary healthcare centers. *Dubai Health Auth. Int. J. Community Med. Public. Health*. **6**, 1–7 (2019).

[11] Goodall, R. J. et al. A systematic review of the impact of foot care education on self-efficacy and self-care in patients with diabetes. *Eur. J. Vasc. Endovasc. Surg.* **60** (2), 282–292 (2020). 32660807 10.1016/j.ejvs.2020.03.053

[12] Centers for Disease Control and Prevention. Clinical guidance for healthcare professionals. Diabetes. (2022). Available from: https://www.cdc.gov/diabetes/hcp/clinical-guidance/index.html

[13] Cohen, J. *Statistical Power Analysis for the Behavioral Sciences* 2nd edn (Academic, 2013).

[14] Singer, A. J., Tassiopoulos, A. & Kirsner, R. S. Evaluation and management of lower-extremity ulcers. *N. Engl. J. Med.* **378** (3), 302–303 (2018). 29342384 10.1056/NEJMc1715237

[15] Bus, S. et al. IWGDF guidance on the prevention of foot ulcers in at-risk patients with diabetes. *Diabetes Metabolism Res. Reviews*. **32** (S1), 16–24 (2016). 10.1002/dmrr.2696 26334001

[16] El-Sedawy, D. & Behairy, A. Impact of preventive diabetic foot nursing intervention on foot status among patients with diabetes. *J. Health Med. Nurs.* **25** (1), 104–114 (2016).

[17] Ali, M. M. & Ghonem, S. E. Effectiveness of health education program regarding foot self-care on risk for developing foot ulcer among patients with diabetes. *Am. J. Nurs.* **8** (5), 280–293 (2019).

[18] Sharoni, S., Abdul Rahman, H., Minhat, H., Ghazali, S. & Ong, M. A self-efficacy education programme on foot self-care behavior among older patients with diabetes in a public long-term care institution, malaysia: A quasi-experimental pilot study. *BMJ Open.* **7**, e014393 (2017). 28600363 10.1136/bmjopen-2016-014393 PMC5623401

[19] Moussa, M. & Gida, N. Effect of foot self-care program among diabetic elderly adults in geriatrics home. *IOSR J. Nurs. Health Sci.* **6** (3), 41–51 (2017).

[20] Grillo, M. et al. Diabetes education in primary care: A randomized clinical trial. *Cad. Saúde. Públ.* **32** (5), 31–39 (2016). 10.1590/0102-311X00097115 27253458

[21] Singh, S., Jajoo, S., Shukla, S. & Acharya, S. Educating patients of diabetes mellitus for diabetic foot care. *J. Family Med. Prim. Care*. **9** (1), 367–373 (2020). 32110620 10.4103/jfmpc.jfmpc_861_19 PMC7014829

