# Supplementary material for: Stepping up: impact of health education on diabetic foot Self-Care in a Resource-Limited tertiary care setting
Source: Sci Rep. 2025 Jul 8;15:24551. doi: 10.1038/s41598-025-08246-1 (PMC12238599; doi:10.1038/s41598-025-08246-1)
Supplement: Supplementary file 1 — Supplementary Material 1 [file 41598_2025_8246_MOESM1_ESM.pdf]

“Stepping Up: Impact of Health Education on Diabetic Foot Self-Care in a Developing Country Tertiary Care Setting”

Serial number:

❖ Sociodemographic data:

1. Age (in years): ………….
2. Sex: ☐ male ☐ female
3. Residence: ☐ urban ☐ rural
4. Educational level: ☐ illiterate ☐ primary education ☐ Secondary education ☐technical education ☐ university
5. Family history of diabetes: ☐ none ☐ yes 1st degree ☐ yes 2nd degree ☐ other
6. Occupation: ☐ not working ☐ working
7. Income: ☐ not enough ☐ enough ☐ enough & saving

❖ Diabetic history:

8. Type of diabetes: ☐type 1 ☐type 2
9. Duration of diabetes (in years) ………
10. Treatment: ☐ Insulin ☐ Oral Hypoglycemic ☐ Both
11. Foot Ulcer: ☐ none ☐ was present and healed ☐currently present
12. Complications of diabetes: ☐No ☐ Neuropathy ☐ retinopathy ☐ Nephropathy ☐ Others
13. Comorbidities: ☐ Hypertension ☐ Renal ☐ cardiac ☐ hepatic ☐ others
14. Regularly monitoring Blood Glucose (at least every month): ☐ No ☐Yes at home ☐ Yes at lab
15. Previously advised about foot self-care: ☐ No ☐ Yes, during the last 3 months ☐ Yes, during the last 6 months ☐Yes, during the last year
16. what is the source? ☐ Media ☐ Physician ☐ nurse ☐ Friend ☐ Family member ☐ others (mention)…………………

❖ Foot Self-care Knowledge:

1. Anti-diabetic medications should be taken:
 regularly irregularly indifferent
2. Feet should be inspected for (cuts, redness, or any change in nails or skin) at least:
 daily every other day weekly
3. Feet should be washed at least:
 daily every other day weekly
4. Feet should be washed using:
 warm water hot water cold water
5. The temperature of water should be checked before use:
 yes no I don't know
6. Soaking feet in water during wash is:
 favorable unfavorable indifferent
7. Feet should be completely dried after washing:
 yes no I don't know
8. To prevent feet dryness, lotion should be applied to:
 top of the foot bottom of the foot both
9. To keep the area between your toes healthy you should:
 use talcum powder use moisturizing cream or Lotion indifferent
10. Shoes should be:
 well-fitting ill-fitting indifferent
11. The inside of shoes should be inspected before wearing them:
 yes no I don't know
12. shoes should be worn:
 with socks without socks indifferent
13. Socks should be changed at least:
 daily every other day weekly
14. Walking barefoot is:
 favorable unfavorable indifferent
15. Shoes or slippers should be worn:
 inside a home outside home both
16. Toenails should be trimmed straight across:
 yes no I don't know
17. To keep blood flowing when you are sitting you should:
 put my feet up put my feet down indifferent
18. If you have redness, blister, wound, or cut on your feet you should:
 consult my doctor at home treatment by OCT drugs
 I don't know

❖ Foot self-care Practices:

Items	Always	often	sometimes	rarely	Ne[illegible]
1. You inspect your feet.					
2. You wash your feet.					
3. You change your socks.					
4. You use lotion or moisturizing cream on your feet.					
5. You use lotion or moisturizing cream between your toes.					
6. You check your shoes before you put them on.					
7. You check your shoes when you take them off					
8. You dry your feet after washing.					
9. You dry between your toes.					
10. You trim your toenails.					
11. You wear sandals.					
12. You wear slippers.					
13. You wear pointed-toed shoes.					
14. You break into new shoes gradually.					
15. You wear artificial fiber (e.g. nylon) socks.					
16. You wear shoes without socks?					
17. You walk outside the house bare feet.					
18. You use a hot water bottle in bed.					
19. You check the water temperature you wash your feet in.					
20. When you get one, You put dry dressing on a blister or wound.					
21. You seek professional help with any problem.					
